# Validity of the Oral Hygiene Ability Instrument (OHAI)

**DOI:** 10.1111/ger.12796

**Published:** 2024-11-06

**Authors:** Ingela Grönbeck Lindén, Marika Wenemark, Pia Andersson, Synneve Dahlin‐Ivanoff, Lars Gahnberg, Catharina Hägglin

**Affiliations:** ^1^ Department of Behavioral and Community Dentistry Institute of Odontology, The Sahlgrenska Academy, University of Gothenburg Göteborg Sweden; ^2^ Centre of Gerodontology, Public Dental Service Gothenburg Sweden; ^3^ Department of Health, Medicine and Caring Sciences, Faculty of Medicine and Health Sciences Linköping University Linköping Sweden; ^4^ Unit for Public Health and Statistics Linköping Sweden; ^5^ Faculty of Health Sciences, Kristianstad University Kristianstad Sweden; ^6^ AgeCap University of Gothenburg Göteborg Sweden; ^7^ Department of Psychiatry and Neurochemistry Institute of Neuroscience and Physiology, University of Gothenburg Göteborg Sweden; ^8^ Department of Oral Diseases Institute of Odontology, Karolinska Institutet Solna Sweden

**Keywords:** construct validity, criterion validity, dental care, frail older adults, oral health

## Abstract

**Objective:**

To evaluate the validity of the newly developed Oral Hygiene Ability Instrument (OHAI), created to assess the cause of any inability of older adults to perform oral hygiene, and to revise the instrument based on the results.

**Background:**

Good oral hygiene is among the most important prophylactic measures for oral health. This applies especially to older adults, among whom risk factors and physical and cognitive barriers are more common and can hamper oral hygiene.

**Methods:**

The OHAI contains (I) an interview, (II) clinical examination, (III) observation of oral hygiene activities and a summarising part. In the study, 149 older adults in three groups participated: stroke, cognitive disorder and general dental patients. Inclusion criteria were to be ≥65 years old, have at least one tooth and to manage oral hygiene without assistance. For criterion validity, sensitivity and specificity were calculated using eight reference instruments. To determine construct validity, we used known group validity, factor analysis and Rasch analysis.

**Results:**

The criterion and construct validity of the OHAI were found to be acceptable to good. In the stroke group, *balance* and *fine motor skills* were assessed to affect oral hygiene most; in the cognitive disorder group, it was *balance*, *coordination*, *spatial ability* and *cognitive functions.* Analyses revealed that one item had no added value and that some response options were not optimal.

**Conclusion:**

The OHAI proved to be valid for the group it is intended for, with only minor revisions needed, resulting in a 32‐item instrument. The OHAI could be a valuable person‐centred tool in prophylactic work with older adults with failing oral hygiene.

## 1 INTRODUCTION

In much of the world, including Sweden, demographics are shifting toward a larger proportion of older adults in the population.^1^, ^2^ The need for medical and dental care in this growing group of older adults is already highly demanding for healthcare services, and this will only increase further. Practical issues surrounding care for older adults can be challenging, and dental care is often a low priority. Older adults, especially if frail, merit attention from dental care before major oral health problems arise.^3^

In dental care, it is relevant to use methods specially adapted to the ageing population.^4^ Older adults usually try to maintain their abilities and habits regarding oral care, but with increasing frailty, help and care regarding oral hygiene are often necessary.^5^ Problems with oral hygiene can be caused by, for example, physical problems (e.g. impaired fine motor skills) or cognitive impairment. If an older adult has poor oral hygiene, it may be useful to have an instrument that assesses what has caused the poor oral hygiene. This information can be used to implement person‐centred measures to remedy the oral health problems.^6^

To strengthen work with older adults in dental practice, we have developed a new instrument, the Oral Hygiene Ability Instrument (OHAI). The development of the OHAI has previously been described.^7^, ^8^ The OHAI was developed to better understand what causes poor ability when it comes to self‐performed oral hygiene for an older adult at the individual level. Examination with the OHAI can give the dental hygienist or dentist a better starting point for individualised decisions about suitable help and support to maintain good oral health.

To our knowledge, no other instrument has been developed to assess the complex activity of maintaining oral hygiene in a salutogenic way – that is, examining the abilities that are present, and helping to address the abilities that are lacking.^9^ The instrument is intended to be used when a change, such as worsened oral hygiene, has been detected in a previously clean mouth, or when a patient visits the clinic for the first time and poor oral hygiene is found.

To be useful, an instrument needs to have good reliability and validity. The OHAI has previously been shown to have an acceptable to good reliability.^10^ The next step in the evaluation is to validate the instrument. The *content validity* of the OHAI has, however, already been investigated in two pilot studies, and proved to be good.^7^ Criterion validity and construct validity remain to be tested. *Criterion validity* can be divided into concurrent and predictive validity.^11^ Concurrent validity refers to the degree to which the scores on a measure are related to another measure established as valid (i.e. a reference instrument), and is used in this study. Predictive validity refers to the ability of a measure to predict a future outcome, and could not be assessed in this study due to a lack of longitudinal data. In *construct validity*, the instrument's scores are assessed according to existing knowledge of the construct.^12^ That is, whether the items of the OHAI are consistent with the hypothesis that oral hygiene may be a complex activity for older adults and can be influenced by the previously identified psychological, functional and environmental dimensions.^8^

The aim of this study was to test the OHAI for criterion validity and construct validity, and, based on the results, to revise items and remove unnecessary items to obtain an improved and user‐friendly final version of the instrument.

## 2 MATERIALS AND METHODS

This cross‐sectional validation study was performed in Region Västra Götaland, Sweden, between 2019 and 2022. The study was approved by the Regional Ethical Review Board in Gothenburg (reference numbers 298‐18 and 2019‐06574) and complied with the World Medical Association's 2013 Helsinki Declaration.^13^

### 2.1 Study sample

The validity of the OHAI instrument was investigated using data from 149 older adults. The inclusion criteria for all groups were being ≥65 years old, having at least one natural tooth or osseointegrated implant, and handling daily oral hygiene without assistance. The study group comprised three clinically separated groups: stroke (n = 50), cognitive disorder (n = 49) and general dental patients (n = 50). The first two groups were intended to represent two types of disabilities/diseases that can affect oral hygiene in different ways, and the third group general older patients in dental care. This group was defined as functionally independent, but high blood pressure, diabetes and heart disease were common in this group.

Information about the study was given to staff at two stroke rehabilitation/training centres for older adults. Interested individuals who had had a stroke contacted the person responsible for the study. Participants with a diagnosis of cognitive impairment were recruited from six nursing homes. The nurse on site received information about the study, asked the residents and, when applicable, their relatives about participation. The general dental patient group was recruited consecutively from a recall list for dental check‐ups at one public dental clinic. Only a few from all groups declined participation, generally due to failing health. During the data analysis, it turned out that one person in the cognitive impairment group did not meet the inclusion criteria and was therefore excluded.

In connection with the invitation, potential participants were informed in writing that participation was voluntary, that they could withdraw from the study at any time, and that confidentiality was guaranteed during data processing. On the day of the interview, each participant also received this information orally and signed a consent form, either themselves or with the help of a relative.

### 2.2 Instruments

#### 2.2.1 The Oral Hygiene Ability Instrument (OHAI)

For the examination with the OHAI, the rater needs a light source, probe and mouth mirror, as well as a study box containing a toothbrush, toothpaste, interdental brushes, hand lotion, a hairbrush and a comb. When the assessment is conducted in the older adult's home, in a nursing home, or in a clinical setting, the participant should preferably brush their teeth in front of a sink and mirror.

The version of the OHAI used in the validation study included 33 items divided into three parts as well as a summarising final part (Table 1):

1. Questionnaire covering background and social context (eight items), dental care and xerostomia (the subjective feeling of dry mouth) (11 items), in total 19 items.
2. Clinical examination (six items)
3. Observation of practical ability to perform oral hygiene (ADL) (eight items)

**TABLE 1:** Content of OHAI items and the raters' assessment “summarising part” in abbreviated form.

Part I. Interview		Part II. Clinical examination	Part III. Observation	Summarising part Raters' assessment
Background, social context	Dental care, xerostomia	Oral function, dental status	ADL, oral hygiene	Impact on oral hygiene of:
1. Medication (names)	9. Regularity of dental care^6^	20. Function: clench jaws^14^	26. Pick up toothbrush^20^	Cognitive function^21^
2. Diseases (names)	10. Who handles contacts with the dental service^7^	21. Function: lick lips^15^	27. Pick up toothpaste^20^	Frailty^21^
3. Disabilities (names), Balance^1^	11. Importance of oral health^8^	22. Function: blow up cheeks^16^	28. Unscrew cap^20^	Motivation^21^
4. Housing^2^	12. Frequency of oral hygiene^9^	23. Mirror test: oral dryness^17^	29. Apply toothpaste^20^	Vision^21^
5. Living conditions^3^	13. Knowledge of oral hygiene^10^	24. Dental status: position of teeth, missing teeth^18^	30. Brush^20^	Fine motor skills^21^
6a. Help with shopping^1^ 6b. Help with personal hygiene^1^	When brushing/picking, how often: 14. Pain^11^ 15. Disgust or gagging^11^	25. Dental status: prosthetics, dentures^19^	31. Pick up interdental Cleaning stick/ brush^20^	Coordination^21^
7. Perceived loneliness^4^	16a. Bleeding when brushing/picking^1^ 16b. If yes, what do you do?^12^		32. Use interdental Cleaning stick /brush^20^	Knowledge of oral hygiene^21^
8. Experienced life‐situation^5^	How often: 17. Problems swallowing dry food^13^ 18. Problems with dry mouth^11^		33. Rinse mouth with water^20^	Spatial ability^21^
				Oral clearance^21^
	19. Problems chewing dry food^13^			Balance problems^21^

Response options:

^1^ Yes/no.

^2^ Own accommodation; Short‐term accommodation; Alternating accommodation (nursing home/at home); Nursing home.

^3^ Lives alone; Lives with a partner (husband, wife, common‐law partner); Lives with children or other relatives; Has a partner but lives separately.

^4^ Yes, often; Yes, every now and then; No; Do not know/no opinion.

^5^ Very satisfied; Quite satisfied; Quite dissatisfied; Very displeased.

^6^ Regularly – at least once a year; Regularly – less often than once a year; Irregularly; Emergency visits only; Not at all.

^7^ Myself; Relative/friend; Care staff; Not relevant, has no dental contact.

^8^ Very important; Quite important; Not particularly important; Not at all important.

^9^ More than twice/day; Twice/day; Once/day; Less often than once/day.

^10^ Very confident; Fairly confident; Quite uncertain; Very uncertain.

^11^ Never; Seldom; Often; Always.

^12^ Proceeds as before; Proceeds more cautiously; Stops.

^13^ Yes, without any difficulty whatsoever; Yes, with some difficulty; Yes, with great difficulty; No, not at all.

^14^ Clear activity is recorded when two fingers are held on the jaw muscles on both sides; Some activity; No activity; The patient does not cooperate.

^15^ Can bring the tip of the tongue along the lips and reach the corners of the mouth; Partially copes with it; Cannot; The patient does not cooperate.

^16^ Can inflate cheeks without leakage of air or making noise; Pass partially (<3 s); Cannot; The patient does not cooperate.

^17^ Glides easily; Glides sluggishly; Does not glide at all.

^18^ Good biting conditions; Single dental gaps and/or broken teeth and/or crowding; Poor bite conditions, many missing and/or broken teeth.

^19^ Own teeth in good condition (also crown/bridge fitted); Own teeth in poor condition; Partial dentures or implants; Full dentures in one or both jaws.

^20^ Copes easily; Copes with some difficulty; Copes with help; Cannot cope at all.

^21^ The degree to which 10 possible influencing factors/conditions cause “no,” “some” or “major” problems for the older adult when managing oral hygiene.

Based on various OHAI items in parts I, II and III, the raters evaluate how 10 possible influencing factors – cognitive function, frailty, motivation, vision, fine motor skills, coordination, knowledge of oral hygiene, spatial ability, oral clearance and balance – affect the participants' oral hygiene ability.

In part I, the rater (a dental professional) interviews the older adult to identify some of the factors that may affect oral hygiene in different ways. These interviews are particularly intended to identify influencing factors such as *frailty*, *motivation* for oral care (including social support/pressure), *knowledge* of oral/dental care and *oral clearance*. The interview can also detect *cognitive impairment*.

In part II, the rater clinically examines the older adult's limitations and resources in terms of oral motor skills, biting conditions, dental status and dry mouth. Part II is also intended to examine *oral clearance* and influencing factors such as *spatial ability* and *cognitive function* (i.e. understanding instructions).

Part III is an observation of the older adult's ADL (Activities of Daily Living) ability regarding oral hygiene. The oral hygiene activity is performed under the rater's instruction and supervision. If possible, the structured activity should take place in the adult's bathroom or at a sink in the treatment room so that environmental aspects such as *balance* and *vision* can be assessed. The activity is also intended to capture *fine motor skills*, *coordination*, *spatial ability*, *motivation*, *frailty* and *cognitive function*.

In the OHAI, the factors that may influence an older person's ability to manage their oral hygiene are to be captured by single or multiple items in parts I, II and III. Which OHAI items (their numbering) are intended to capture each factor/condition can be seen in Table 4 further on, and Table 1 presents the meanings of the different items.

After the rater completes the three parts of the OHAI, their next task is to rate in an assessment box the degree to which 10 possible influencing factors/conditions cause “no,” “some” or “major” problems for the older adult when managing oral hygiene (Table 1). The summarising part is the basis for the actions that are decided on together with the older adult and others concerned.

#### 2.2.2 Reference instruments

There is no other single instrument that can be used as a reference for the entire OHAI instrument. Criterion validity was therefore tested by comparing items intended to capture different factors/conditions affecting oral hygiene ability against relevant validated reference instruments. Those instruments are described below.

Cognitive impairment was defined as ≤24 points on the Mini Mental Test (MMSE‐SR) scale.^14^

The Modified Schirmer Test (MST)^15^ was used as a measure of unstimulated salivary flow. Salivary secretion was measured with a saliva indicator on a coloured paper strip after 3 minutes: ≥25 mm = no dry mouth; <25 mm = dry mouth. Hue‐Check Gum (Hue)^16^ was used to measure chewing function. This test includes evaluation of chewing efficiency by judging the colour mixture and bolus formation of two different coloured gums using the Subjective Assessment Scale (SA): SA1 = chewing gum not mixed, impressions of cusps or folded once; SA2 = much of the chewing gum unmixed; SA3 = bolus slightly mixed; SA4 = bolus well mixed; and SA5 = bolus perfectly mixed. In this study, SA4 and SA5 were regarded as “no problem” with chewing function. The Nine‐Hole Peg Test (NHPT)^17^ was used to measure the dexterity of the dominant hand. Cut‐off values according to gender, age and time to complete the test were used: satisfactory fine motor skills = within the normative values; unsatisfactory fine motor skills = outside the normative values. Hand strength was measured using a North Coast hand dynamometer (NC)^18^ for the dominant hand as a measure of weakness. Proposed cut‐off values according to gender and age were used. The Timed Up and Go (TUG)^19^, ^20^ instrument was used to assess balance during sitting, rising, walking and sitting down again. The procedure was clocked, and cut‐off values for locomotion time (in seconds) were applied as follows: ≤10 seconds and ≤11‐20 seconds were regarded as no problems with balance; ≤21‐29 seconds = partly dependent on walking aid, variety in locomotion; >30 seconds = dependent on support of a carer or a wheelchair. To measure frailty, the four‐item FRESH instrument^21^ was used, and participants were considered at risk of frailty if they answered “yes” to two or more of the four items. Visual acuity was measured using the KM chart at a distance of 1.5 m.^22^, ^23^ Visual impairment was defined as a visual acuity of ≤0.5 using both eyes and the commonly used visual aid.

### 2.3 Data collection

Most examinations took place at a dental clinic or in a room at a nursing home, but 46 assessments were conducted in participants' homes. For all participants, especially those in the cognitive disorder group and the stroke group, a relative or healthcare staff member were present at the examination if desired.

Data from the OHAI and from the reference instruments were collected separately. The OHAI items were read to the participant and the form was completed by the rater. First, the OHAI was administered by a dental professional and, after a short break, the tests with the reference instruments were administered by one of the authors (IGL). Five raters were involved in the OHAI data collection: two dental hygienists, two dental nurses and one dentist. The raters were trained in the test procedure before the data collection started and were also given access to the written OHAI manual. The person performing the OHAI assessment was unaware of the results of examinations with the reference instruments and vice versa.

For most items, data from 147 to 149 participants were registered, unless stated otherwise in the tables. An exception is item 20 in part II, “bite hard,” for which data are missing for 40 participants. Several raters found this item difficult to assess and refrained from assessing it. Item 20 was therefore not included in the validity analysis. In the summarising part of the raters' assessment of vision, 122 examinations were missing. This was discovered too late, so vision was not included in the analyses of the raters' summarising assessment part.

### 2.4 Statistical analysis

Data management and analysis were carried out in SPSS version 26.0 (IBM Corporation, Armonk, NY) and Rumm 2030+.^24^ Descriptive statistics are presented in frequencies (n), percentages (%), means, standard deviations (SD) and ranges. Percent agreement (PA) was used for agreement in one analysis of factors/conditions. When multiple OHAI items were used in the validation process, these were dichotomised and then summed, or, as in the case of part III items, all response option scores were summed. If the sum was zero, this meant that the older adult was unaffected by the factor/condition, whereas ≥1 meant that the older adult was affected.

For *criterion validity*, sensitivity/specificity was used. *Construct validity* was evaluated in terms of known group validity, factor analysis and Rasch analysis. Known group differences between the three study groups were analysed using the Kruskal–Wallis (KW) test and the Mann–Whitney (MW) *U*‐test as post hoc tests, and the full ordinal and continuous scales were used in the analyses. Factor analysis with principal components and varimax rotation was used for part I (items 9, 11‐19). A Rasch analysis was conducted on part III of the OHAI to evaluate the response options (signs of unordered thresholds) and investigate the potential to generate a valid scale for oral hygiene ability in a larger patient sample in the future. Spearman's correlations were used for correlation between the raters' assessment of the factors'/conditions' influence on oral hygiene (OH) and OHAI items/sums of items. Statistical significance was considered at *p* < 0.05 for all analyses.

## 3 RESULTS

### 3.1 Participants

The socio‐demographic characteristics of the 149 participants are shown in Table 2. Some significant differences were found between the groups, especially regarding living in a nursing home and whether or not participants were living alone, but not in regard to gender (Table 2).

**TABLE 2:** Sociodemographic data for the study participants for the stroke, cognitive disorder and patient groups and in total.

	Stroke	Cognitive disorder	General patients	Total
Number of participants, n	50	49	50	149
Gender, female, %	46	67	64	59
Age, mean (SD)^a^	79.8 (8.6)	84.8 (6.9)	77.3 (6.8)	80.6 (8.1)
Age, range	66‐100	70‐97	65‐90	65‐100
Housing: nursing homes, %^d^	46	84	2	44
Living conditions: with partner, %^e^	42	18	64	42
Number of medications, mean (SD)^b^	5.0 (3.9)	2.9 (3.9)	3.4 (3.5)	3.7 (3.9)
Number of diseases, mean (SD)^c^	2.0 (1.2)	1.7 (0.9)	1.5 (1.2)	1.7 (1.2)

^a^ Kruskal–Wallis: *P* < 0.001; post hoc: cognitive > stroke (*P* = 0.004); cognitive > general (*P* < 0.001).

^b^ Kruskal–Wallis: *P* < 0.001; post hoc: stroke > general (*P* < 0.001); stroke > cognitive (*P* = 0.012).

^c^ Kruskal–Wallis: *P* = 0.012; post hoc: stroke > general (*P* = 0.006); stroke > cognitive (*P* = 0.037).

^d^ Chi‐squared: *P* < 0.001; post hoc: cognitive > stroke (*P* < 0.001); cognitive > general (*P* < 0.001); cognitive > stroke (*P* < 0.001).

^e^ Chi‐squared: *P* < 0.001; post hoc: cognitive < stroke (*P* < 0.001); cognitive < stroke (*P* = 0.009); stroke < general (*P* = 0.022).

### 3.2 Criterion validity

Table 3 presents the criterion validity in the form of sensitivity and specificity. Very good sensitivity for the OHAI's purposes was found for the OHAI *frailty* items, particularly for the sum of the part I items 4, 6a/b and 10, relative to the reference instruments FRESH and TUG. Also, part III items 28‐33 capturing *fine motor skills* displayed good sensitivity relative to the reference instrument NC, as did part II's sum of function/status items 21‐22, 24 and 25 relative to the reference instrument Hue capturing problems with *oral clearance*. The strongest sensitivity was for *balance* problems captured by item 3 relative to the reference instrument TUG, but with low specificity. *Vision* displayed questionable criterion validity with both low sensitivity and low specificity. The other items/sums of items displayed acceptable sensitivity/specificity for their purposes relative to their reference instruments.

**TABLE 3:** Percentages of participants assessed to have problems according to the reference instruments. The OHAI items and agreement in terms of sensitivity and specificity in relation to the reference instruments. Percentages in brackets have already been presented in another row of the table.

n = 147‐149 unless stated otherwise	Proportion with problem		Agreement	
	Reference %	OHAI %	Sensitivity %	Specificity %
Cognitive function				
MMSE – Part I: items 2 and 3	30	30	68	86
MMSE – Part III: sum of items 26‐33^a^	30	62	52	84
Frailty				
FRESH – Part I: sum of items 4, 6a/b, 10	58	63	83	65
FRESH – Part III: sum of items 26‐33^a^	(58)	(62)	76	54
TUG – Part I: sum of items 4, 6a/b, 10	72	63	81	85
TUG – Part III: sum of items 26‐33^a^	(72)	(62)	75	73
Motivation – no reference instrument				
Part I: sum of items 5, 7‐9, 11, 12	‐	26	‐	‐
Vision				
KM – Part I: sum of items 27, 31, 32	33	48	26	41
Fine motor skills				
NHPT – Part III: sum of items 28‐33^a^	78	57	64	69
NC – Part III: sum of items 28‐33^a^	33	(57)	79	55
Coordination				
NHPT – Part III: sum of items 28‐30, 32^a^	77	56	63	68
Knowledge – no reference instrument				
Part I: sum of items 9, 12, 13	‐	29	‐	‐
Spatial ability – no reference instrument				
Part III: sum of items 30, 32^a^	‐	49	‐	‐
Oral clearance				
MST – Parts I, II: sum of dry mouth items 17, 18, 22^a^	64	63	66	43
HUE^b^ – Part II: sum of function/status items 21‐22, 24, 25^a^	72	79	82	45
Balance problems				
TUG – Part I: item 3	72	74	85	64
FRESH – Part I: item 3	58	(74)	69	72
Emotions – no reference instrument				
Part I: sum of items 14‐16	‐	24	‐	‐

^a^ These OHAI items are also intended to capture other factors/conditions in the OHAI. The percentage therefore does not necessarily mean that the factor/condition listed is the one that the participant has a problem with.

^b^ For the reference instrument HUE, six participants did not want (or dare) to chew on the chewing gum, n = 143.

### 3.3 Construct validity

#### 3.3.1 Known group validity

Analysing known group differences revealed whether the differences between groups were those expected considering the particular groups and each factor/condition, which was found to be largely the case (Table 4). The stroke group had more problems with the factor/condition *balance* and to some extent with *fine motor skills* and *oral clearance*. The cognitive disorder group had more problems in terms of *motivation*, *knowledge*, and especially *cognitive function*, *frailty* and *spatial ability*. In the general dental patient group, a low percentage was assessed to have problems for all factors/conditions except balance. For the dry mouth items in the factor *oral clearance*, and for the items intended to capture *emotions*, there were no statistically significant differences between groups (Table 4).

**TABLE 4:** Known group validity between the stroke, cognitive disorder and general dental patient groups and the percentage with problems for each of the factors/conditions distributed by group.

	Stroke (n = 50)	Cognitive disorder (n = 49)	General patients (n = 50)	Kruskal–Wallis	Post hoc: Mann–Whitney *U*‐test			
	Problems	Problems	Problems		Stroke > General	Cognitive > General	Stroke > cognitive	Cognitive > stroke
	%	%	%	*P*	*P*	*P*	*P*	*P*
Cognitive function								
Part I: items 2 and 3	8	84	0	<0.001	0.042	<0.001		<0.001
Frailty								
Part I: sum of items 4, 6a/b, 10	78	94	18	<0.001	<0.001	<0.001		0.004
Part III: sum of items 26‐33	70	82	34	<0.001	<0.001	<0.001		
Motivation (n = 146)								
Part I: sum of items 5, 7‐9, 11, 12	82	89	51	<0.001	0.001	<0.001		
Vision								
Part I: sum of items 27, 31, 32	58	67	20	<0.001	<0.001	<0.001		
Fine motor skills								
Part III: sum of items 28‐33	64	78	30	<0.001	<0.001	<0.001		
Coordination								
Part III: sum of items 28‐30, 32	64	74	30	<0.001	<0.001	<0.001		
Knowledge								
Part I: sum of items 9, 12, 13	34	40	14	0.011	0.021	0.003		
Spatial ability								
Part III: sum of items 30, 32	52	69	26	<0.001	0.001	<0.001		0.007
Oral clearance								
Parts I, II: sum of dry mouth items 18, 19, 23	72	56	54	0.075				
Part II: sum of function/status items 21‐22, 24, 25	72	69	64	0.310				
Balance problems								
Part I: item 3	96	75	50	<0.001	<0.001	0.009	0.004	
Emotions								
Part I: sum of items 14‐16	36	22	14	0.060				

As a further validation of the OHAI items/sums of items used to capture the different factors/conditions, the reference instruments were also analysed for known group differences (data not shown). The results were very similar, especially in that the stroke group and the cognitive disorder group were more affected than the general dental patient group. However, there were some minor differences between the stroke group and the cognitive disorder group that did not appear in the OHAI item analyses. For *frailty*, the FRESH instrument showed that the stroke group was more affected than the cognitive disorder group, while items chosen to capture *frailty* showed that the cognitive disorder group was more affected. Regarding *fine motor skills*, both the NHPT and NC instruments showed that the stroke group was more affected than the cognitive disorder group, while this was not captured by the OHAI items.

#### 3.3.2 Factor analysis

From part I, 10 items (items 9, 11‐19) were investigated using factor analysis. The factor analysis generated three factors with eigenvalues of 1.69, 1.53 and 1.40, which together explained 54% of the variance. The factors were: 1. Routines for dental care (items 9, 11‐13), 2. Oral function (items 17‐19) and 3. Problems when brushing and picking the teeth (items 14‐16) (Table 5).

**TABLE 5:** Shows the rotated component matrix (rotation converged in five iterations) for the three factors in part I (items 9, 11‐19).

Component			
OHAI items Part I	1	2	3
9. Regularity of dental care	–0.661	−0.105	−0.076
11. Importance of oral health	0.677	−0.011	−0.021
12. Frequency of oral hygiene	0.609	−0.107	0.154
13. Knowledge of oral hygiene	0.650	0.044	−0.013
14. Bleeding when brushing/picking	0.004	0.022	0.784
15. Pain when brushing/picking	0.129	−0.037	0.804
16. Disgust or gagging when brushing/picking	−0.010	0.424	0.485
17. Problems swallowing dry food	0.122	0.783	0.078
18. Perceived dry mouth	−0.180	0.758	0.036
19. Problems chewing hard food	0.465	0.520	−0.140

Extraction method: principal component analysis.

Rotation method: varimax with Kaiser normalisation.

#### 3.3.3 Rasch analyses

The analysis showed that the Rasch model fit the items in part III (items 26‐33) reasonably well. The Rasch analysis showed clear signs of disordered thresholds due to problems with the response options “copes easily,” “copes with some difficulty,” “copes with help” and “can't cope at all.” A change in the response options in part III from four to three options (combining response options “manage with some difficulty” and “manage with help”) improved the model.

### 3.4 The raters' summary assessment part

After the three parts of the OHAI were completed, the raters assessed the degree to which the 10 possible influencing factors, validated above, caused problems for older adults in managing oral hygiene. Correlations between the raters' assessment of the degree to which the factors/conditions had affected oral hygiene and the OHAI items intended to capture the specific factors/conditions were moderate or strong for all factors except *motivation* (Table 6).

**TABLE 6:** Proportions of study participants assessed by the raters as having problems managing oral hygiene due to the proposed factors/conditions, and the correlation between the raters' assessment of the factors'/conditions' influence on oral hygiene (OH) and the OHAI items/sums of items intended to capture these factors/conditions; n = 147‐149.

	Raters' assessment impact on OH %	Spearman's rank correlation rho
Cognitive function	17	
Part I: items 2 and 3		0.36
Part III: sum of items 26‐33		0.53
Frailty	20	
Part I: sum of items 4, 6a/b, 10		0.43
Part III: sum of items 26‐33		0.46
Motivation	7	
Part I: sum of items 5, 7‐9, 11, 12		0.20
Vision – no analysis due to missing data		
Fine motor skills	37	
Part III: sum of items 28‐33		0.52
Coordination	28	
Part III: sum of items 28‐30, 32		0.57
Knowledge	13	
Part I: sum of items 9, 12, 13		0.38
Spatial ability	22	
Part III: sum of items 30, 32		0.50
Oral clearance	25	
Part I, II: sum of dry mouth items 17, 18, 22		0.45
Part II: sum of function/status items 21‐22, 24, 25		0.37
Sum of all the dry mouth and function/status items		0.47
Balance problems	34	
Part I: item 3		0.55

Analysis of differences between groups showed that the stroke group was most affected by the factors/conditions *fine motor skills* and *balance*, and to some extent by *oral clearance* (Table 7). The cognitive disorder group was more affected by the factors/conditions *frailty*, *motivation*, *coordination*, *knowledge*, *spatial ability*, and especially *cognitive function* and *knowledge*. In the general dental patient group, apart from balance, a low percentage were assessed to have problems with oral hygiene due to any of the factors/conditions.

**TABLE 7:** Proportions of study participants in the stroke, cognitive disorder and general dental patient study group assessed by the raters to have moderate or major problems with their oral hygiene due to the respective factors/conditions, and the construct validity of known group differences between the groups.

Raters' assessment	Stroke (n = 50)	Cognitive disorder (n = 49)	General patients (n = 50)	Kruskal–Wallis	Post hoc: Mann–Whitney *U*‐test			
Impact on oral hygiene of	Problems	Problems	Problems		Stroke^a^ > General patients	Cognitive^a^ > General patients	Stroke^a^ > Cognitive	Cognitive^a^ > Stroke
	%	%	%	*P*	*P*	*P*	*P*	*P*
Cognitive function	18	33	0	<0.001	0.002	<0.001		
Frailty	26	31	4	<0.001	0.002	<0.001		
Motivation	2	18	0	<0.001		0.002		0.007
Fine motor skills	66	35	10	<0.001	<0.001	0.003	0.005	
Coordination	32	45	6	<0.001	0.001	<0.001		
Knowledge	10	29	2	<0.001		<0.001		0.021
Spatial ability	28	37	0	<0.001	<0.001	<0.001		
Oral clearance	36	31	8	0.003	0.001	0.005		
Balance problems	94	78	28	<0.001	<0.001	<0.001	0.001	

^a^ Indicating the more affected study group.

## 4 DISCUSSION

This study has shown that the validity of the newly constructed Oral Hygiene Ability Instrument (OHAI) is mainly good in this sample of older adults. Validating an instrument is crucial in order to know that the instrument truly measures what it is intended to measure. However, it should be borne in mind that validation is a process, not an outcome: validation continues with the use of the instrument, which also applies to this newly developed one.^25^

### 4.1 Strengths and limitations – method

To our knowledge, the OHAI is the first instrument to take into account the complexity that oral hygiene presents to older adults. In the current study, the validity of the OHAI has been thoroughly investigated using several different methods depending on the type of validity to be assessed and on which method was considered best suited for the different parts of the OHAI, its items and the factors/conditions in question. However, a weakness was that some of the reference instruments used to assess criterion validity were found not to be optimal during the course of the study. This was most evident for the KM chart with the purpose of assessing *vision*, but there were also limitations for the Modified Schirmer Test (MST) for assessing the dry mouth aspect of *oral clearance* and for the Nine‐Hole Peg Test (NHPT) for capturing *fine motor skills* and *coordination*.

The low sensitivity and specificity of the KM chart for *vision* is likely due to the fact that it is a simple examination, which only measures central vision, that is, the vision used when, for example, watching TV. For oral hygiene, it is also necessary to be able to see fine details up close. The factor *vision* is nevertheless important to take into account, as dental care providers should recommend older adults to use their visual aids when performing oral hygiene.

For the reference instrument MST, the sensitivity to the summed dry mouth items was low. This lack of agreement between the criterion items can probably be explained. Self‐perceived dry mouth and hyposalivation can be seen as two separate conditions. Their prevalence varies widely, and it is not common to see both conditions in the same individual.^26^ Better values would probably have been obtained if a dry mouth instrument other than the MST had been used, but this would have been too time consuming and labour intensive for the participants. It probably would have been preferable if MST had been used in a more standardised way, for example in the morning before eating and talking, but this was not feasible. Questions about dry mouth are often used, but with questionable results^27^ However, it is important to keep these self‐reported questions in the OHAI together with the clinical mirror test (item 23, part II) to capture whether dry mouth is perceived as a problem for the older adult and be able to provide relevant advice.

Of the two reference instruments used to examine *fine motor skills*, namely the NHPT for dexterity and the NC for grip strength, the NHPT for some reason displayed somewhat low sensitivity and specificity. This was also the case for the NHPT in relation to *coordination*. However, dexterity has previously been found to be of the greatest importance for being able to perform oral hygiene.^28^ Grip strength, often used as a measure of frailty, is also important for performing oral hygiene, but it is subordinate to dexterity when it comes to this ability.^29^, ^30^

*Cognitive ability* was captured partly by the two anamnestic items 2 and 3 and partly by the observational part III. The two anamnestic questions in relation to the reference instrument Mini Mental Test (MMSE‐SR) provided acceptable sensitivity, which could probably be improved by a minor change in the OHAI. The problem with the somewhat low sensitivity is not the reference instrument itself but rather that these two items are not specifically about cognitive impairment but ask about diseases (item 2) and disabilities (item 3). If a specific question about cognitive impairment is asked instead, the sensitivity will probably increase. The sensitivity was also low for the observational part III related to the MMSE; this was unsurprising and could be because this part is meant to capture not only cognitive impairment but also other influencing factors/conditions like *fine motor skills* and *coordination*.

### 4.2 Validity of the OHAI

To assess criterion validity, the agreement between the OHAI items and the respective reference instruments was calculated using sensitivity and specificity. As the OHAI is designed to detect impaired ability, it might be reasonable to prioritise sensitivity over specificity, as over‐identification is better than not detecting an older adult who is on the verge of losing independence.^31^ The OHAI items on *balance*, *oral clearance* and *fine motor skills* showed good sensitivity to the reference instruments used. The *frailty* items showed both good sensitivity and good specificity. With the exception of the vision items, the criterion validity of the remaining items was acceptable despite some reference instruments not being optimal. This indicates that to assess the factors/conditions that affect an older adult's oral hygiene ability, the OHAI can work well, and it is easier to administer the single OHAI instrument than several different specific instruments.

Construct validity was analysed using known group validity, factor analysis and a Rasch analysis. The differences between the examined groups that emerged in the known group analysis were very much as expected. The stroke group had more difficulties with *balance* and the cognitive disorder group had more problems with *cognitive function*, *frailty* and *spatial functions*. *Frailty* was expected to be more prevalent in the cognitive disorder group because most participants in this group lived in nursing homes and were significantly older than the participants in the other two groups. However, frailty was also very common in the stroke group.

When it comes to *fine motor skills*, the stroke group was affected, but the cognitive disorder group had even more such problems, which is reasonable as dexterity is affected by neurocognitive disorders. It is also a fact that *fine motor skills* decline with age for everyone, and the cognitive disorder group was significantly older than the other groups.

*Oral clearance* is the ability to clear the oral cavity of food residues and avoid retaining and pocketing food after a meal.^32^ This important function depends on several abilities; for example, saliva secretion, biting, chewing and using the muscles of the mouth and tongue. Feeling food residue or food stuck between the teeth requires good sensory skills (e.g. oral stereognosis).^33^ Regarding *oral clearance* and the known group analysis, the differences between the groups were not statistically significant for the dry mouth items, but for the oral function items, the stroke group and cognitive disorder group had more problems than the general dental patient group. This was expected, considering that several of these functions deteriorate over the years and are affected by illness such as stroke and by cognitive function.^34^, ^35^, ^36^

For cognition, group analysis showed that the cognitive disorder group performed oral hygiene better than the stroke group (part III). Habits formed early in life often persist for a long time, and even if one is cognitively affected, one can still manage oral hygiene thanks to learned patterns. The stroke patients, however, had suffered an injury that disrupted this pattern both physically and psychologically.^37^

In part I, the sum of items 14‐16 was merged into a new influencing factor: emotions.^38^ No differences between the groups were found in terms of emotions, which was expected, since emotions such as fear of pain, bleeding or gagging seldom are affected by illness but are something that only certain people suffer from. Not many respondents in this study stated that they had problems with these emotions, but the factor/condition could influence oral hygiene for those affected and it is therefore important to capture. Unpleasant emotions can lead to avoidance behaviour regarding oral hygiene, and this can easily be overlooked by dental professionals, as some patients may not be inclined to admit that they experience fear or discomfort associated with something as natural as cleaning their teeth.

In addition, items 9 and 11‐19 in part I were subjected to *factor analysis*, and three factors emerged: “oral hygiene routines,” “problems when cleaning teeth” and “oral function.” Most items loaded highly on one factor group, but two items (i.e. items 15 and 19) loaded relatively high on two factor groups. Item 15, “gagging/disgust,” can be explained by gagging being a problem related to both cleaning teeth and oral function. For item 19, “trouble chewing hard food,” the problem is usually avoided by abstaining from hard food, which may become a routine. The three factors that were generated in the factor analysis indicated that the selected 10 items from the interview part (part I) were relevant to what they were intended to investigate.

The items of part III are well suited for developing a unidimensional scale of oral health ability, since they represent different levels of difficulties and abilities. Therefore, a preliminary Rasch analysis was conducted even though the study group in this validation study was small and also contained a number of healthier older adults. The targeting of the stroke group and the cognitive disorder group seems promising, and in the next step of the OHAI development, a digital version with automatically generated individual scores for oral health ability would improve usability of the instrument.

### 4.3 The raters' summary assessment part

The last part of the validation study deals with the degree to which the 10 possible influencing factors caused problems for the older adults in maintaining oral hygiene. This was examined through raters' assessments of the 10 influencing factors. The correlations calculated for these factors were moderate to good, except in the case of *motivation*. Motivation is linked to willingness to perform.^38^ In this study, very few respondents were unwilling to attempt the tasks they were asked to do, which is quite natural because they had volunteered. This may have affected the results for the influence of motivation on oral hygiene.

The correlation values were very much as anticipated, as the aim of the OHAI items was to capture each specific factor/condition, whereas the rater's assessment would capture whether the factor/condition affects oral hygiene, so stronger associations would have been questionable.

The difference between the groups was as expected: being in the general dental patient group had less influence on the ability to perform oral hygiene than being in the cognitive disorder or stroke group. Notably, being in the cognitive disorder group had the greatest influence on the ability to perform one's own oral hygiene.

Balance problems were common in all groups and were assessed to greatly affect the ability to perform oral hygiene in both the stroke group and the cognitive disorder group. Balance is not usually considered in this context but is important as it can influence the ability to perform oral hygiene.^39^ If the bathroom environment is not adapted for an older adult who has poor balance or is in a wheelchair, oral hygiene can be affected. For example, one may need a chair and mirror at an appropriate height, or sufficient space so a wheelchair can access the sink. This shows that part III of the OHAI should be observed in the respondent's bathroom or at a sink in the clinic to get a natural view of the toothbrushing situation. If that is not possible, then the anamnestic questions about illness and disability (part I, items 2 and 3) could identify the balance issue and lead to follow‐up questions.

The factors found to be influencing oral hygiene ability are meant to be captured in many ways by the OHAI, not least in the interview part, which can function as conversation support where the questions can be expanded on, for example, if an answer is short or only yes/no.

### 4.4 Changes in the OHAI following the evaluation

After evaluating the OHAI, minor changes will be necessary to obtain a robust instrument. In this study, it was found that the two anamnestic items (i.e. items 2 and 3) need to be reformulated to better capture specific problems with functional impairments, such as cognitive impairment and balance problems. In part II, the clinical examination, item 20 on oral function (i.e. bite hard) proved difficult to assess by several raters, and produced many missing assessments. It was therefore decided to omit this item. The other two items (i.e. items 21 and 22) on oral functioning were considered sufficient to capture oral function in the *oral clearance* factor. The OHAI will then contain 32 items.

The influencing factors *fine motor skills* and *coordination* showed a very high percentage of agreement (97%) in the raters' assessment. Thus, *fine motor skills* and *coordination* will be merged into one influencing factor. Items 14‐16 in part II, meant to capture fear of bleeding, pain and gagging, formed a new influencing factor, *emotions*, that is to be added to the raters' assessment part.

The Rasch analysis clearly showed that one of the four answer options in part III did not add anything; instead, the model was improved when the middle two answer options were combined. These minor changes may also result in a more user‐friendly instrument. Although the raters found the OHAI easy to use, any changes that lead to improvements and facilitate the use of the instrument by both dental professionals and older adults can be beneficial.

### 4.5 Future perspectives

A digital version of the OHAI, using modern technologies such as artificial intelligence, is in the process of being developed. The handling of the OHAI can then be simplified and the raters' subjective assessment can be reduced in terms of the information collected from the OHAI. If the OHAI is implemented in dental care, it will add an important tool to the prophylactic arsenal. The OHAI is then likely to prove useful for other people than older adults when poor oral hygiene is detected. Professional groups other than dental personnel, such as occupational therapists and assistant nurses in home care and nursing homes, will probably also make use of the OHAI in the future. Part III of the OHAI – observation of toothbrushing activity – is easy to use and can quickly provide a lot of information. In certain situations, it may therefore be possible and appropriate to use only this part to quickly determine whether it is *cognitive function*, *fine motor skills/coordination*, *spatial ability* or *vision* that affects the ability to manage oral hygiene.

That oral hygiene is multifactorial in nature should be investigated further, for example, in different cultures, ages or how different socio‐economic aspects affect oral hygiene. For such studies, the OHAI is well suited.

## 5 CONCLUSION

The OHAI is a person‐centred assessment instrument for use in prophylactic work to analyse the cause of impaired oral hygiene in older adults. It is now available for both clinical use and research. The instrument has previously been shown to have good reliability; this evaluation in addition proved the OHAI to be valid for the purpose it is intended for, with only minor revisions needed.

## AUTHOR CONTRIBUTIONS

IGL administered and handled the data collection. IGL and CH were responsible for conceptualisation design, analysis and interpretation of data. MW was involved in interpretation and analysis of data. IGL wrote the original draft of the manuscript with support from CH. MW, PA, SDI and LG were involved in the design of the study and in critically reviewing the manuscript. All authors approved the published version.

## FUNDING INFORMATION

The Local Research and Development Council Gothenburg and Södra Bohuslän. The Swedish order of Freemasons, Grand Lodge of Sweden. The Royal Society of Arts and Science in Gothenburg. Agneta Prytz‐Folke and Gösta Folkes Foundation, Gothenburg.

## CONFLICT OF INTEREST STATEMENT

The authors declare no conflict of interests.

## Data Availability

Data are available from the corresponding author upon reasonable request.
